# Surface Plasmon Resonance Based on Molecularly Imprinted Polymeric Film for l-Phenylalanine Detection

**DOI:** 10.3390/bios11010021

**Published:** 2021-01-15

**Authors:** Duygu Çimen, Nilay Bereli, Adil Denizli

**Affiliations:** Department of Chemistry, Hacettepe University, Beytepe, 06800 Ankara, Turkey; duygucimen@hacettepe.edu.tr (D.Ç.); bereli@hacettepe.edu.tr (N.B.)

**Keywords:** l-phenylalaine, surface plasmon resonance, sensor, molecular imprinting technology, detection

## Abstract

In this study, we designed a simple, rapid, sensitive and selective surface plasmon resonance (SPR) sensor for detection of L-phenylalaine by utilizing molecular imprinting technology. l-phenylalanine imprinted and non-imprinted poly(2-hydroxyethyl methacrylate-methacryloyl-l-phenylalanine) polymeric films were synthesized onto SPR chip surfaces using ultraviolet polymerization. l-phenyalanine imprinted and non-imprinted SPR sensors were characterized by using contact angle, atomic force microscopy and ellipsometry. After characterization studies, kinetic studies were carried out in the concentration range of 5.0–400.0 μM. The limit of detection and quantification were obtained as 0.0085 and 0.0285 μM, respectively. The response time for the test including equilibration, adsorption and desorption was approximately 9 min. The selectivity studies of the l-phenylalanine imprinted SPR sensor was performed in the presence of d-phenylalanine and l-tryptophan. Validation studies were carried out via enzyme-linked immunosorbent analysis technique in order to demonstrate the applicability and superiority of the l-phenylalanine imprinted SPR sensor.

## 1. Introduction

l-phenylalanine (l-Phe), which is an aromatic and hydrophobic amino acid, is particularly effective in the treatment of brain diseases as it can cross the blood–brain barrier. l-Phe is the precursor amino acid of l-tyrosine (l-Tyr) and is involved in several protein-related activities [1,2,3]. l-Phe is used by the brain as a precursor to produce dopamine and nor-epinephrine, which provide transmission between the brain and nerve cells. Phenylketonuria (PKU) can be treated and caused by deficiency of phenylalanine hydroxylase enzyme in newborn babies [4,5]. In the case of late diagnosis, neurological functional disorders, major behavior ral disorders, autism and eczema can occur by PKU. It is also a disease that is the most important cause of mental retardation [6,7]. This results in a deficiency in the conversion of phenylalanine to tyrosine and increased levels of other metabolites and phenylalanine. The measurement of the phenylalanine (Phe) concentration in blood and urine is important in genetic studies and treatment of PKU [8,9,10]. Although the safe PKU range in plasma is 120–360 µM, doctors trying for the lower portion of this range. For that reason, the desired PKU levels in the plasma is 21–137 μM for infants and children and 35–85 μM for adults [11,12]. For the diagnosis of PKU disease, phenylalanine, known as an indicator substance in the diagnosis of PKU disease, has different methods, including the amount of plasma determining Guthrie test, microbial inhibition, fluorimetric, chromatography and mass spectrometry [13,14,15,16]. However, these methods are time consuming and expensive, insufficient sensitivity, complex instrumentation, special laboratory facilities and highly experienced labor force requirements limit their use in routine analysis. Simple, fast and economical methods of determination are needed for the diagnosis of the PKU disease.

The molecular imprinting method is an important method with special cavities for identification of target molecules in a polymeric matrix [17]. Stable synthetic polymers with selective molecular recognition regions are prepared by the molecular imprinting technology. With the molecular imprinting method, selective and unique artificial receptors belonging to a specific target molecule can be designed. The molecular imprinted method proposes a selective, sensitive, reusable, high stability, low cost and easy-to-use approach for the detection, adsorption, identification and separation of molecules [18]. One of the application areas of molecular imprinting method is the use of surface plasmon resonance (SPR) sensors as recognition elements [19]. SPR sensors can analyze the binding of target molecules to be determined on a SPR sensor chip surface coated with a metal film (gold, 50 nm) directly without any labeling. One of the advantages of SPR sensors is thanks to their sensitive refractive index changing during the surface binding, giving the results simultaneously and label free. SPR sensors are used in various fields for food quality control analysis [20,21], environmental analysis [22] and diagnostic purposes in medicine [23,24,25,26].

In this study, l-phenylalanine (l-Phe) imprinted and non-imprinted poly(2-hydroxyethyl methacrylate-methacryloyl-l-phenylalanine) SPR sensor for l-phenylalanine (l-Phe) detection from aqueous solution and artificial plasma was prepared by utilizing molecular imprinting technology. The selectivity of the l-Phe imprinted SPR sensors was evaluated with competitive agents such as d-phenylalanine (d-Phe) and l-tryptophan (l-Try). Validation studies were carried out via the enzyme-linked immunosorbent analysis (ELISA) technique in order to demonstrate the applicability and superiority of the prepared SPR sensor.

## 2. Materials and Methods

### 2.1. Materials

l-phenylalanine, d-phenylalanine, l-tryptophan, 2-hydroxyethyl methacrylate (HEMA), ethylene glycol dimethacrylate (EGDMA) and allyl mercaptan (CH_2_CHCH_2_SH) were delivered from Sigma-Aldrich (St. Louis, MI, USA). *N*,*N*′-azobisisobutyronitrile (AIBN) and other utilized chemicals were purchased from Merck AG (Germany). Artificial human plasma was supplied by Tokra Medical (Ankara, Turkey). SPR bare gold chips (SPRchip^TM^, Masidon, WI, USA) were supplied for the SPRimager II instrument by GWC Technologies (Masidon, WI, USA).

### 2.2. Preparation of l-Phenylalanine Imprinted and Non-Imprinted SPR Chip

The gold surface of the SPR chip was modified with an allyl mercaptan in previous articles [23]. The SPR chip was cleaned with a piranha solution (3:1 concentrated H_2_SO_4_:H_2_O_2_, *v*/*v*) and then washed with ethanol–water mixture. After cleaning, the SPR gold chip was incubated in allyl mercaptan to introduce allyl groups onto the surface of the SPR chip at room temperature. After, the SPR chip surface was washed with absolute ethly alcohol and ultrapure water. l-Phe imprinted and non-imprinted polymer film onto allyl modified SPR gold chip was carried out by UV polymerization method.

For the preparation of l-phenylalanine (l-Phe), imprinted and non-imprinted poly(2-hydroxyethyl methacrylate-methacryloyl-l-phenylalanine) polymeric film onto SPR sensor at different template: monomer (l-Phe:MAPA) ratios (1:1, 1:2, 1:3, 1:4 M ratio) were synthesized onto allyl mercaptan modified SPR chip surface. ΔR and imprinting factor (IF) values were calculated for l-Phe imprinted (MIP) and non-imprinted (NIP) polymeric films. The imprinting factor was calculated as follows:

The imprinting factor (IF): ∆R_(MIP_)/∆R_(NIP)_.

**Figure 1:**
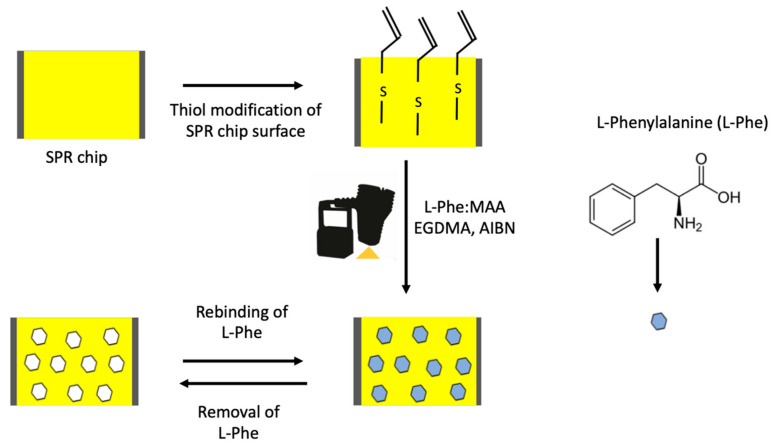
Schematic illustration of the preparation of the l-phenylalanine (l-Phe) imprinted surface plasmon resonance (SPR) sensor.

In the first step, the pre-complex was prepared with the model molecule l-Phe (8.0 mg) and N-methacryloyl l-phenylalanine (MAPA, 6.9 μL) monomer in 1:3 molar ratio at room temperature for 2 h. The synthesis procedure of the MAPA functional monomer was reported in our previous study [26,27]. The prepared pre-complex was mixed with HEMA (6.1 μL), EGDMA (56.5 μL). After, the AIBN (2 mg) was added to this monomer mixture. Then, 5 μL of this solution was dropped onto the SPR gold chip surface using a spin coater. It was then allowed to polymerize under UV light (100 W, 365 nm). The non-imprinted polymeric film onto the allil mercaptane modified SPR chip gold surfaces of SPR chip were prepared without l-Phe (Figure 1).

### 2.3. Characterization Studies

Characterization studies of unmodified, imprinted and non-imprinted SPR chips were used with contact angle measurements, atomic force microscopy and ellipsometer. Contact angle measurements were performed for hydrophilicity characterization of unmodified, imprinted and non-imprinted SPR chips surface by using the KRUSS DSA100 (Hamburg, Germany) device. To determine the hydrophobicity of SPR chip surfaces, the sessile drop method was used [28]. Three different photographs were taken by dropping water on different parts of the SPR chip surface and the mean of contact angle values were obtained. Morphology of SPR chip surfaces was obtained using an atomic force microscope (Nanomagnetics Instruments, Oxford, UK) in the tapping mode [29]. The samples of 1 × 1 μm^2^ area were taken in the 1 μm/sec scanning speed and 256 × 256 pixel resolution. The thicknesses of polymeric films onto the SPR sensor surface were determined by using an auto-nulling imaging ellipsometer measurement (Nanofilm EP3, Germany) [30]. All thickness measurements were performed at an incidence angle of 62° at a wavelength of 532 nm and were made at six different points on the SPR chip surface. Thickness measurements of the SPR chip surface were repeated three times and the results were reported by averaging these values.

### 2.4. Kinetic Analysis

Real time detection of l-Phe from the aqueous solution was performed using l-Phe imprinted and non-imprinted SPR sensors. Firstly, the l-Phe imprinted SPR sensor was washed with deionized H_2_O, then pH 7.4 phosphate buffer was applied to the SPR system. Aqueous solutions of l-Phe with various concentrations (5.0–400.0 μM) were investigated by the SPR system. % change in reflectivity (%ΔR) of SPR sensor was monitored in all kinetic analysis in the SPR sensors. Removal of l-Phe bounded onto SPR sensor surface was used with 0.1 M NaCI as desorption agent for 10 min. After the desorption step, the l-Phe imprinted SPR sensor was cleaned with deionized water and pH 7.4 phosphate buffer. The equilibration–adsorption–regeneration cycles were performed for all kinetic studies. The SPR kinetic analysis was performed for l-phenylalanine using SPR imager II (GWC Technologies, Masidon, WI, USA). In this study, the SPR sensor system was used in the Kretchmann configuration and the angle of incident light measured at which surface plasmon resonance takes place. During the study, flow rate was 150 μL/min (0.031″ ID tubing); operating wavelength 800 nm; and prism material SF10 glass [31].

Surface plasmon resonance (SPR) sensors are a simple and direct method based on refractive index changing that occurs very near between probe and thin metal film surface. The refractive index difference between two transparent media and usually gold or silver thin metal film on the middle of these two media enable optical sensing. Above a critical angle, a plane-polarized light beam entering the higher refractive index medium (glass prism) can undergo total internal refraction. At this point, the evanescent wave light that is a component of the electromagnetic field penetrates the gold film. The interaction between the evanescent wave and gold film generates excitation of surface plasmons with free oscillating electrons. As a result, the reflected light intensity is decreased. This photophysical process is the underlying logic of SPR sensors. SPR sensors offer the advantage of rapid, label-free simultaneous monitoring of binding events due to refractive index changes near the SPR sensor surface. SPR images that showed all changes at the SPR chip surface provide detailed information on molecular binding, interactions or kinetic processes. A typical SPR sensor chip is a glass chip coated with a thin layer of chemically inert metal, usually gold [32].

In order to detect the adsorption isotherm parameters, SPR sensors were examined by using Langmuir, Freundlich and Langmuir–Freundlich isotherm models.

The linearized forms of equations for SPR sensors are given as follows;

Langmuir   ∆R = ∆R_max_ C/KD + C (1)

Freundlich   ∆R = ∆R_max_ C ^1/n^ (2)

Langmuir–Freundlich   ∆R = ∆R_max_ C^1/n^/KD + C^1/n^ (3)

∆R is the resonance frequency change. The concentration of l-Phe is C (μM). KA (μM) and KD (1/μM) are the association and dissociation equilibrium constants, respectively. The Freundlich exponent is described with 1/n. The equilibrium, maximum and experimental subscripts refer eq, max and ex, respectively [33,34,35,36].

To determine the selectivity of the l-Phe imprinted and non-imprinted SPR sensors, d-Phenylalanine, (d-Phe) and l-tryptophan (l-Try) were used as competitive molecules. These competitive molecules, which are close to l-Phe in terms of both molecular weight and structure, are used in the selective studies. For selectivity studies, l-Phe imprinted and non-imprinted SPR sensors were determined by applying at 50 μM of each competitive molecules. The selectivity coefficients and relative selectivity coefficients were calculated for the l-Phe imprinted (MIP) and non-imprinted (NIP) SPR sensors. The selectivity and relative selectivity coefficients are k and k’, respectively [36]. The selectivity coefficient (k) is described by the following equations:

k = ∆R_template_/∆R_competitor_ (4)

k’ = k_MIP_/k_NIP_ (5)

### 2.5. Clinical Analysis

The reproducibility and selectivity of the l-Phe imprinted SPR sensor were performed with artificial plasma. Artificial plasma was diluted with 10 mM phosphate buffer (pH 7.4) in the ratio of 1/5 (*v*/*v*). The aqueous solutions of l-Phe at a concentration of 50 and 100 μM were spiked in the diluted artificial plasma 1/5 (*v*/*v*). Firstly, l-Phe imprinted SPR sensor was equilibrated with 10 mM phosphate buffer (pH 7.4) for kinetic analysis. The spiked l-Phe artificial plasma samples were applied to the SPR system. The removal of l-Phe molecules onto l-Phe imprinted SPR sensors was carried out with 0.1 M NaCI solution. Kinetic analysis results of SPR sensors were compared with the results obtained from ELISA measurements performed in Hacettepe University, Ankara, Turkey.

## 3. Results

### 3.1. Characterization Studies

**Figure 2:**
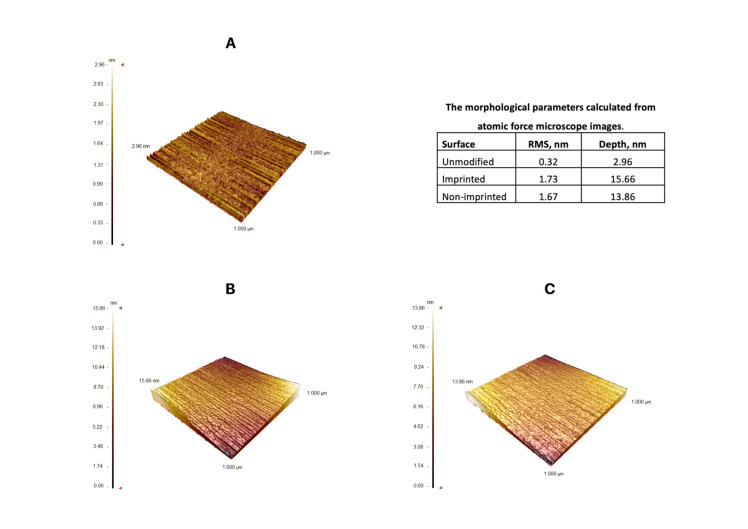
AFM images of SPR sensor surfaces (**A**: bare gold SPR surface, **B**: l-Phe imprinted SPR sensor, **C**: non-imprinted SPR sensor).

**Figure 3:**
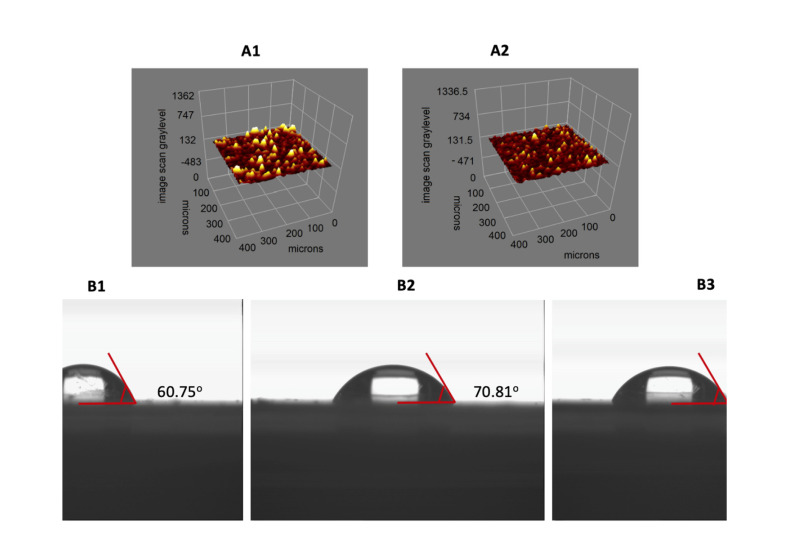
Morphology of bare gold surfaces and l-Phe imprinted SPR sensor surfaces (Ellipsometry images (**A1**: l-Phe imprinted SPR chip, **A2**: non-imprinted SPR chip); contact angles (**B1**: bare gold SPR surface, **B2**: l-Phe imprinted SPR chip, **B3**: non-imprinted SPR surface)).

The surface morphologies of SPR chip surfaces were characterized with atomic force microscobe (AFM) in the tapping mode. The surface deepnesses values of SPR chip surface were 2.96, 15.66 and 13.86 nm for unmodified, l-Phe imprinted and non-imprinted SPR chip surface, respectively (Figure 2). Root mean square (RMS) values of the gold surface were also determined. RMS value of the unmodified gold SPR chip surface was determined as 0.32 nm. After the polymerization process, RMS values of endotoxin imprinted and non-imprinted SPR chip surfaces increased to 1.73 nm and 1.67 nm, respectively. AFM images indicate that polymeric film was homogenously created onto the SPR chip surfaces. The polymeric film thicknesses of SPR chip surface were obtained as 50.4 nm and 46.4 nm for l-Phe imprinted and non-imprinted SPR chip surfaces, respectively (Figure 3A1,A2). According to ellipsometry and AFM results, it is seen that polymeric film is synthesized homogeneously onto SPR chip surfaces.

Contact angle values of unmodified, l-Phe imprinted and non-imprinted SPR chip surfaces were obtained as 60.75°, 70.81° and 64.44°, respectively (Figure 3B1–B3). The observed increase in contact angles depended on the increase of hydrophobicity of SPR chip surfaces. The increase of hydrophobicity of SPR chip surface is expected due to the hydrophobic functional groups of the MAPA monomer.

### 3.2. Real Time Detection of l-Phe with SPR Sensor

**Figure 4:**
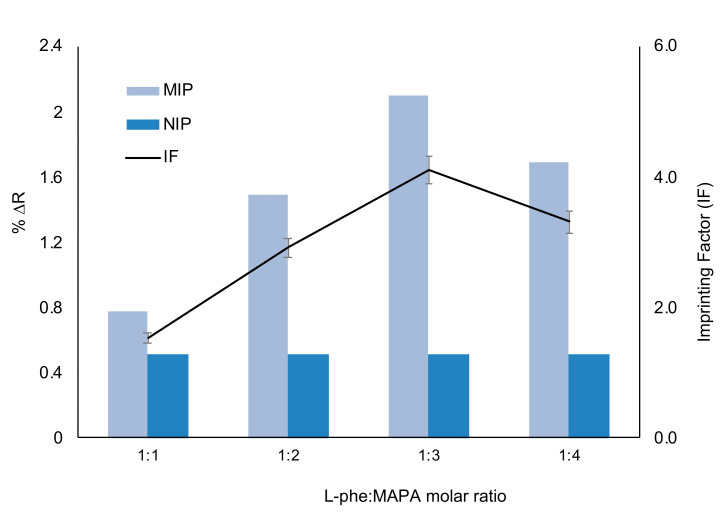
The imprinting factor of l-Phe imprinted and non-imprinted polymeric films.

The imprinting factors at different molar ratios of l-Phe: MAPA were determined for the preparation of l-Phe imprinted and non-imprinted polymeric film in Figure 4. It was showed that the acquired %ΔR value of l-Phe imprinted polymeric films was higher than the non-imprinted polymeric films. l-Phe molecules are successfully located on binding sites molecular cavities according to the results. Maximum imprinting factor value was observed at 1:3 molar ratio of l-Phe: MAPA because of the potential stoichiometric ratio.

**Figure 5:**
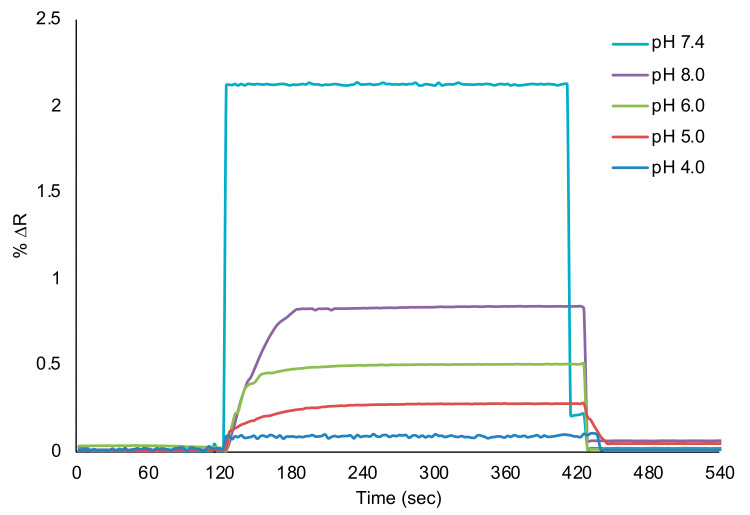
pH analysis for l-Phe imprinted SPR sensors.

**Figure 6:**
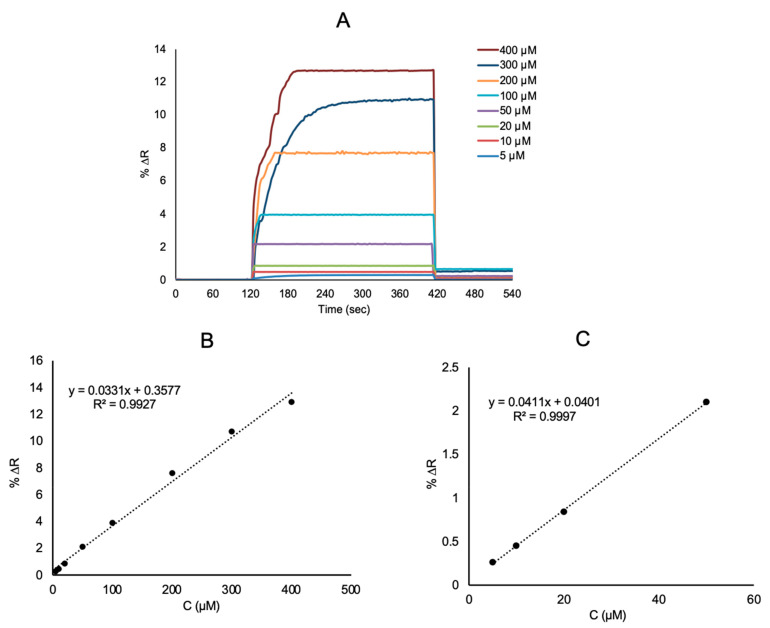
Real-time responses: (**A**) linear regions between 5–400 μM (**B**) and 5–50 μM (**C**) of SPR sensors aqueous solutions of l-Phe at different concentrations.

Kinetic studies were carried out using acetate–phosphate buffers at different pH ranges (pH 4.0–8.0). Aqueous solutions of l-Phe were applied to the SPR system in 50 μM of l-Phe for pH analysis in Figure 5. As seen in Figure 5, pH 7.4 phosphate buffer was chosen as the working medium for the detection of l-Phe in kinetic analysis. According to the kinetic analysis results, the decrease in the sensogram signal was determinative in other buffer systems. It is known that the pH value of the medium affects the complexation reaction between l-Phe and MAPA functional monomer.

l-Phe imprinted and non-imprinted SPR sensors were analyzed with real time l-Phe detection from aqueous solutions and artificial plasma. Firstly, l-Phe imprinted SPR sensor was stabilized with pH 7.4 phosphate buffer as equilibration buffer. After, l-Phe imprinted SPR sensor was performed with aqueous l-Phe solutions in different concentrations of 5.0–400 μM prepared with pH 7.4 phosphate buffer (Figure 6A). These cycles for a new kinetic analysis were realized in 9 min for l-Phe detection. With the interaction of l-Phe solutions and SPR sensors, kinetic data were calculated using SPRview software. As the l-Phe concentration increased, the %ΔR value increased (Figure 6B). The linear requation in the linear range of 5.0 to 400 μM concentration is y = 0.0331x + 0.3577 with a coefficient of determination of 0.992. The limit of detection (LOD = 3.3 S/m) and the limit of quantification (LOQ = 10 S/m) were calculated from the slope of the calibration curve of l-Phe molecules. The standard deviation of the intercept is S and the slope of the regression line is m [37,38]. The LOD and LOQ for l-Phe detection with l-Phe imprinted SPR sensors were 0.0085 μM and 0.0285 μM, respectively. The comparative sensor studies in the literature on the determination of l-Phe are given in the Table 1.

Adsorption isotherm models are used to determine the homogeneity of the l-Phe imprinted SPR sensor. Therefore, Langmuir, Freundlich and Langmuir–Freundlich isotherm models are applied to the l-Phe imprinted SPR sensor. The Langmuir isotherm model is more suitable for l-Phe imprinted SPR sensor among the others (Figure S1). Table S1 presents the all mentioned isotherm models that applied for l-Phe imprinted SPR sensor. The Langmuir model with ΔR_max_ equals 53.19 is found to be the most appropriate model to identify the interaction between l-Phe molecules and SPR sensor. These result shows that the l-Phe binding properties on the prepared SPR sensor surface is monolayer, equivalent energy and lowest lateral interaction and homogenously distributed (Table S1).

**Figure 7:**
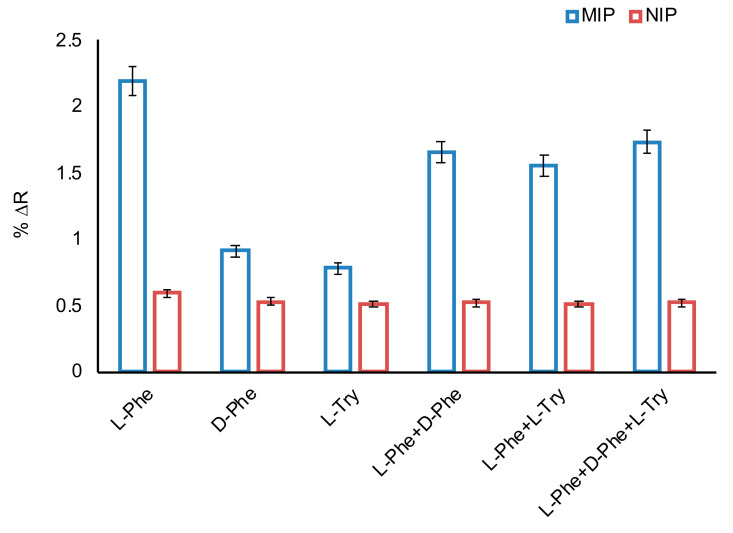
The selectivity studies and comparison of selectivity of SPR sensors (C: 50 μM in all measurements).

To determine selectivity of the imprinted sensor, a non-imprinted sensor was also prepared. Selectivity studies of l-Phe imprinted and non-imprinted SPR sensors were performed with competitive molecules such as d-phenylalanine, (d-Phe) and l-tryptophan (l-Try). These competitive molecules, which are close to l-Phe in terms of both molecular weight and structure are used in the selective studies. For selectivity studies, l-Phe imprinted and non-imprinted SPR sensors were kept constant as 50 μM in phosphate buffer (pH 7.4) of each competitive molecule (Figure 7). l-Phe imprinted SPR sensor was 2.31 and 2.71 times more selective for l-Phe than d-Phe and l-Try, respectively (Table 2). The results showed that the l-Phe imprinted (MIP) SPR sensor had higher adsorption capacity for l-Phe in comparison to d-Phe and l-Try due to selective cavities in the polymeric film on sensor surface. To compare the selectivity of the l-Phe imprinted sensor, a non-imprinted sensor was also prepared and the response (∆R) of the non-imprinted sensor to l-Phe, d-Phe and l-Try was obtained as 0.59, 0.53 and 0.51, respectively. According to the results, the non-imprinted (NIP) SPR sensor had less selectivity than the MIP sensor to l-Phe, due to a lack of l-phenylalanine selective cavities. The relative selectivity coefficient (k’) indicates the affinity of the l-Phe repressed recognition regions. According to the relative selectivity coefficients, the MIP sensor can recognize l-Phe as 2.08 times more selective than d-Phe; and 2.34 times more selective than l-Try, which includes indole ring. Additionally, it can be said that the prepared l-Phe MIP sensor is an effective sensor in the recognition of the d and l-enantiomers of the phenyl alanine amino acid. The fact that the other competitor amino acid tryptophan, which is a hydrophobic amino acid, is not structurally compatible with l-Phe specific cavities in the imprinted sensor with the indole ring it contains, may explain the higher selectivity. As can be seen from the results, the molecular imprinting process brought high selectivity to the prepared sensor.

### 3.3. Clinical Analysis

**Figure 8:**
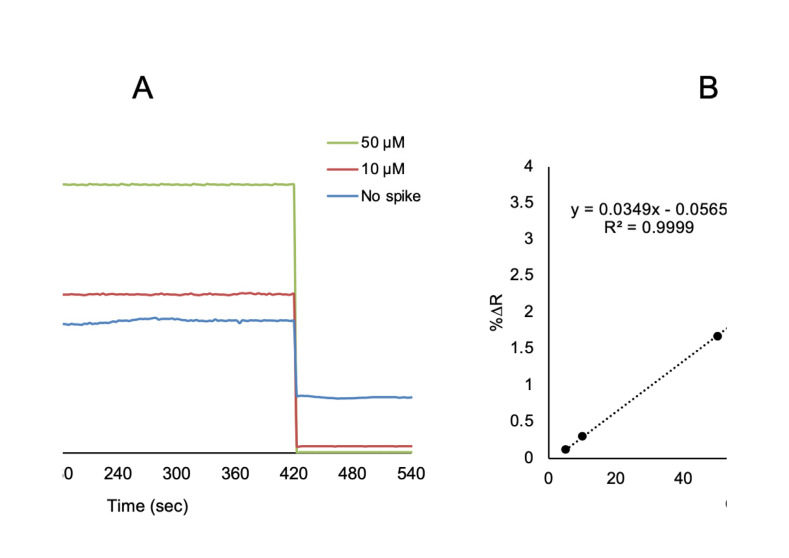
Sensorgram for real-time l-Phe detection (**A**) and calibration curve (**B**) of different l-Phe concentrations (1.0–100 μM) in 1/5 diluted artificial plasma.

To show the reproducibility and practicality of the prepared l-Phe imprinted SPR sensors in the clinical analysis, a different concentration of l-Phe was spiked to artificial plasma samples. The aqueous solutions of l-Phe at a concentration from 10 μM to 50 μM were spiked in the diluted artificial plasma 1/5 (*v*/*v*). Firstly, the l-Phe imprinted SPR sensor was washed with deionized H_2_O, then pH 7.4 phosphate buffer was applied to the SPR system for 2 min. After, artificial plasma without spiked l-Phe was applied to the SPR system. Then, artificial plasma samples spiked with l-Phe at a concentration of 10 μM and 50 μM were applied to the SPR system for 5 min (Figure 8A). Removal of l-Phe bounded onto SPR sensor surface was used with 0.1 M NaCI as desorption agent for 3 min. The % change in reflectivity (%ΔR) of SPR sensor was monitored in all kinetic analysis in the SPR sensors. The linear requation in the linear range of 5.0 to 100 μM concentration was y = 0.0349x − 0.0565 with a coefficient of determination of 0.999 (Figure 8B). To determine the reliability and accuracy of SPR sensor and ELISA, the recovery was calculated (%) (Table 3).

The limit of detection (LOD = 3.3 S/m) and the limit of quantification (LOQ = 10 S/m) were calculated from the slope of the calibration curve of l-Phe molecules. The limit of detection (LOD) and quantification (LOQ) were calculated as 0.172 and 0.573 μM, respectively. The compatibility of the obtained ELISA results with the SPR sensor results indicates that SPR sensor provides reliability, and is accurate, quantitative and sensitive for the determination of l-Phe in artificial plasma samples.

### 3.4. Reusability

**Figure 9:**
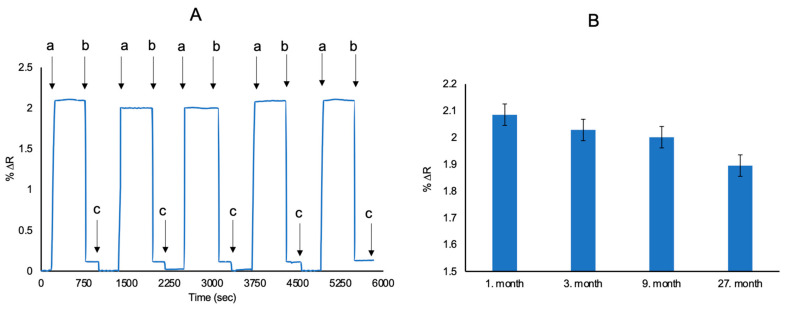
Reproducibility of l-Phe imprinted SPR sensors (**A**) and short-term and long-term stability of l-Phe imprinted SPR sensors (**B**) (C_L-Phe_: 50 μM in all measurements) (equilibration (a), adsorption (b) and desorption (c)).

The most important advantages of SPR sensors are the stability of their polymeric structures, resistance to environmental conditions and longer repeatability. In order to investigate the reproducibility of l-Phe imprinted SPR sensors, l-Phe aqueous solutions were given 5 times to the SPR system (Figure 9). The reproducibility of l-Phe imprinted SPR sensors was examined for l-Phe at 50 μM concentration by using the same SPR sensor. The efficiency value of the SPR sensors were identified as 90.88%. To show storage stability, the l-Phe imprinted SPR sensors were also tested in different times. After 27 months, the efficiency of l-Phe imprinted SPR sensor was 88% in the presence of 50 μM l-Phe. The reusability results suggested that the prepared SPR sensors have high reproducibility and may have value in clinical applications. Statistically evaluated experiments were performed and repeated five times in intraday studies. Reproducability with high precision is shown by standard deviation (SD) and relative standart deviation calculations (RSD%) which are 0.036 and 1.76, respectively. The accuracy is lower than two percent according to intraday RSD% value and this proves that the method can obtain the desired high sensitivity [24].

## 4. Conclusions

Phenylalanine (l-Phe) is the most important disease of treatable mental retardation that results from phenylalanine hydroxylase enzyme deficiency in newborn babies. Therefore, l-Phe play a role in the diagnosis of the phenylketonuria (PKU) disease. In this study, rapid, reliable, accurate, quantitative, sensitive and label-free SPR sensors were developed for the determination of l-Phe in artificial plasma. The limit of detection and quantification for l-Phe in the linear range of 5.0–400 μM were achieved as 0.0085 and 0.0285 μM, respectively. Langmuir, Freundlich and Langmuir–Freundlich isotherm models can be applied to determine the homogeneity of l-Phe imprinted polymeric film onto the SPR sensor. In addition, the prepared imprinted SPR sensor showed a good recognition capacity for the l-Phe compared with other structurally similar molecules. The reproducibility of l-Phe imprinted SPR sensors were identified as 90.88%. Finally, the experimental results demonstrated that l-Phe imprinted SPR sensors can be employed in the quantitative determination of l-Phe in clinical applications.

## Figures and Tables

**Table 1:** The comparison of the prepared sensor system in the literature for determination of l-phenylalanine (l-Phe).

Sensor Transducer	Linear Range	Real Sample	Limit of Detection (LOD)	Ref.
Optical biosensor	10–10,000 μM	-	5–10 μM	[39]
Electrochemical sensor	0.5–100 μM	blood plasma	0.001 μM	[40]
Electrochemical enantiorecognition biosensors	0.001–10,000 μM	plasma	0.001 μM	[41]
Chemiluminescence	0.015–0.12 μM	pharmaceutical and soft drinks	0.00024 μM	[42]
A carbon nanosphere electrode (CN)	1–100 μM	pharmaceutical products	-	[43]
Electrochemical biosensor	0.5–15,000 μM	human blood and saliva	0.416 μM	[44]
Whole-Cell siosensors	5−50 μM	-	4.87 μM	[45]
Chemiluminescence sensor	0.00005–0.5 μM	human plasma	0.000014 μM	[46]
Aptasensor	0.72 μM–6 μM	human plasma	0.23 μM	[47]
Electrochemical sensor	0.01–0.1 μM	egg white and chicken	0.001 μM	[11]
Surface plasmon resonance sensor	5–400 μM	artificial plasma	0.172 μM	This study

**Table 2:** The selectivity and relative selectivity coefficients for competitive molecules for surface plasmon resonance (SPR) sensors.

	MIP Sensor		NIP Sensor		
	∆R	k	∆R	k	k’
l-Phe	2.11	−	0.59	−	−
d-Phe	0.91	2.31	0.53	1.11	2.08
l-Try	0.78	2.71	0.51	1.16	2.34
l-Phe+d-Phe	1.66	1.27	0.52	1.13	1.11
l-Phe+l-Try	1.55	0.59	0.51	1.04	0.56
l-Phe+d-Phe+l-Try	1.73	0.45	0.52	0.98	0.46

Resonance frequency change: ∆R, Molecularly imprinted polymer: MIP, Non-imprinted polymer: NIP, Selectivity coefficients: k, Relative selectivity coefficients: k’, l-phenylalanine: l-Phe, d-Phenylalanine: d-Phe, l-tryptophan: l-Try.

**Table 3:** The recoveries of l-Phe in artificial plasma samples (n:4).

Added l-Phe (μM)	Found l-Phe (μM)		Recovery (%)	
	SPR Sensor	ELISA	SPR Sensor	ELISA
10	10.18 ± 0.005	9.79 ± 0.005	101.87 ± 0.005	97.80 ± 0.049
50	49.24 ± 0.008	48.72 ± 0.005	98.48 ± 0.016	97.46 ± 0.010

l-Phe: l-phenylalanine; SPR: Surface Plasmon Resonance; ELISA: enzyme-linked immunosorbent analysis.

## Data Availability

Not applicable.

## Supplementary Materials

The following are available online at https://www.mdpi.com/2079-6374/11/1/21/s1, Figure S1: Langmuir (A), Freundlich (B) and Langmuir-Freundlich (C) adsorption models for SPR sensors, Table S1: Isotherm parameters for SPR sensors.
