# Supplementary material for: Surface Plasmon Resonance Based on Molecularly Imprinted Polymeric Film for l-Phenylalanine Detection
Source: Biosensors (Basel). 2021 Jan 15;11(1):21. doi: 10.3390/bios11010021 (PMC7830203; doi:10.3390/bios11010021)
Supplement: Supplementary file 1 [file biosensors-11-00021-s001.pdf]

Supplementary material

Surface plasmon resonance based on molecularly imprinted polymeric film for L-phenylalanine detection

Duygu Çimen, Nilay Bereli, Adil Denizli*
Hacettepe University, Department of Chemistry, Beytepe, Ankara, Turkey

Table S1. Isotherm parameters for SPR sensors.

**Langmuir**	**Freundlich**	**Langmuir-Freundlich**
ΔR_{max} : 53.19	ΔR_{max}: 147.94	ΔR_{max} : 42.35
K_D (1/μM) : 0.365	1/n : 0.9183	1/n : 0.9183
K_A (μM) : 2.743	R^2 : 0.933	K_D (1/μM) : 4.475
R^2 : 0.984		K_A (μM) : 0.173
		R^2 : 0.953

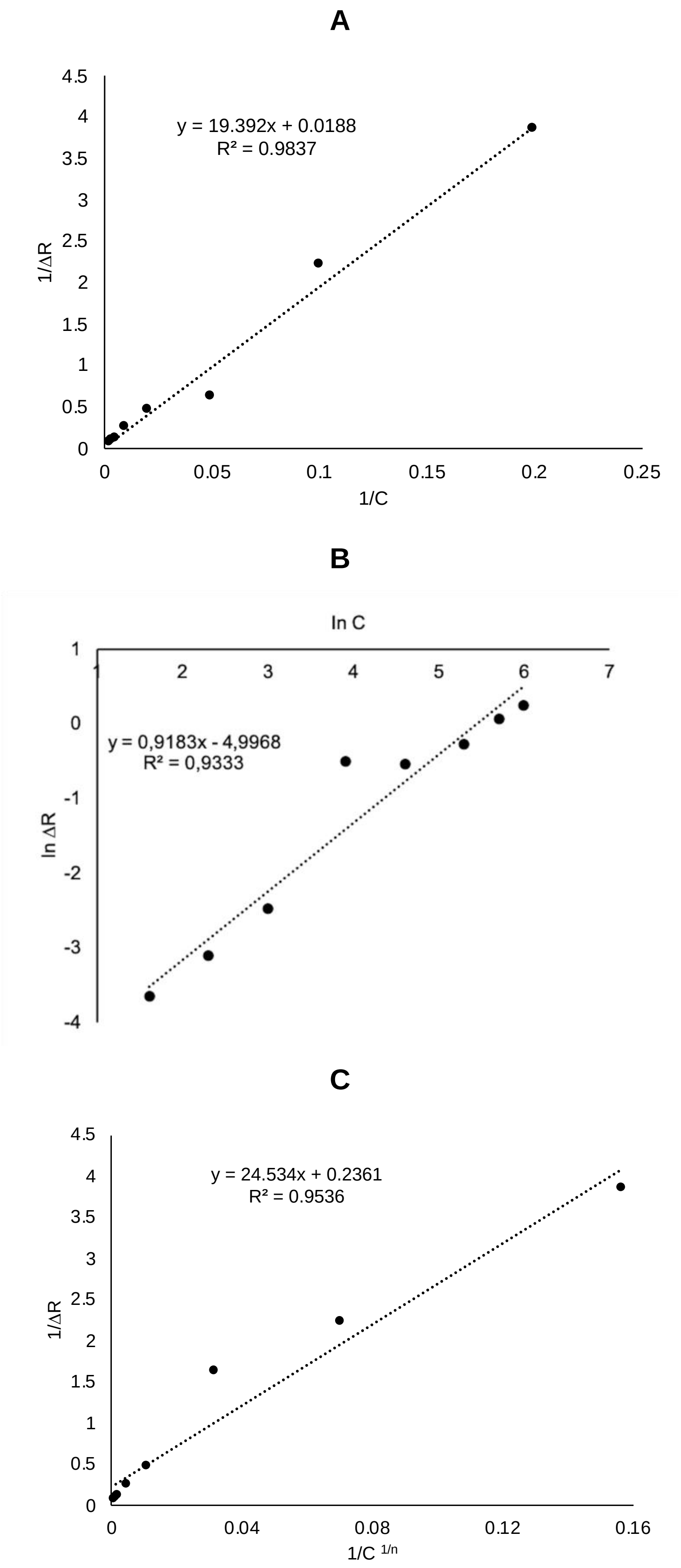

Fig S1. Langmuir (A), Freundlich (B) and Langmuir-Freundlich (C) adsorption models for SPR sensors.

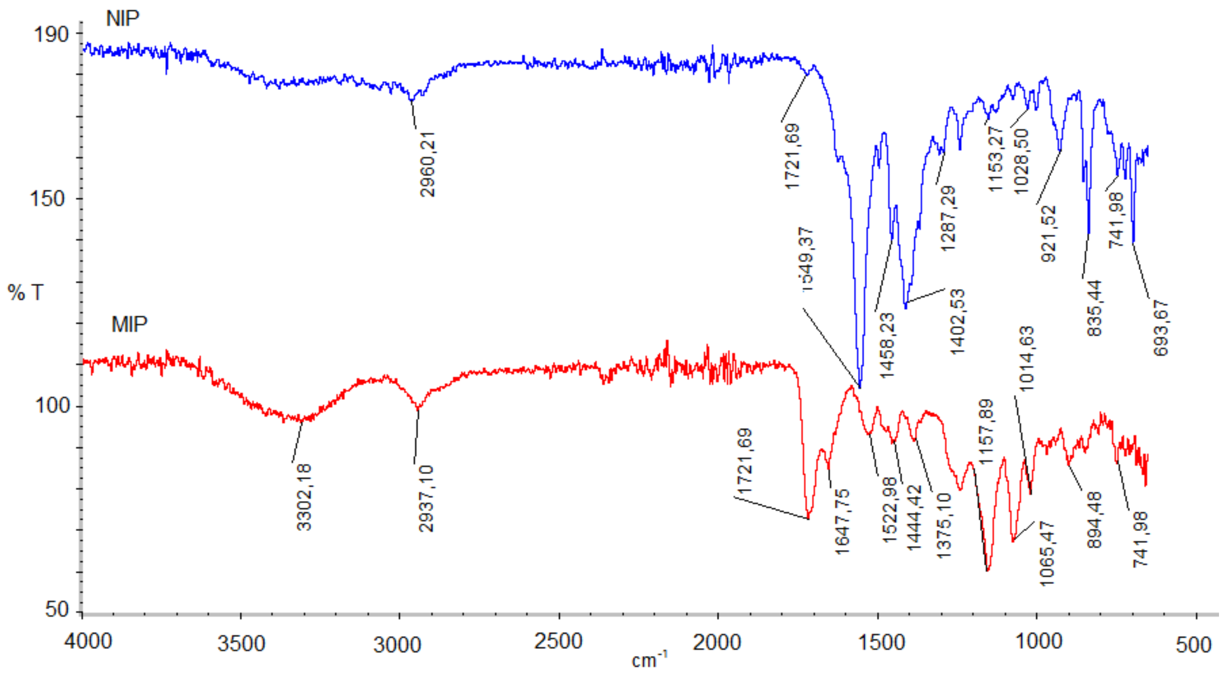

Fig S2. FTIR spectrums of non-imprinted (NIP) and L-Phe imprinted (MIP) polymer coated SPR sensor surface.

The FTIR spectrums were obtained in the frequency region 4000-500 cm^{-1} (FTIR 8000 Series, Shimadzu, Japan). At FTIR-ATR spectrums of non-imprinted (NIP) and L-Phe imprinted (MIP) polymeric films were determined in Figure S2. The incorporation of MAPA monomer into the polymer structure was confirmed with the FTIR spectrum, which contains strong aromatic C-H stretching band at 1025 cm^{-1} and aromatic C-C stretching band around 1500 cm^{-1} [1]. The characteristic bands at 3300 cm^{-1} and 1444 cm^{-1} due to NH asymmetric and COO- stretching confirmed the existence of amino group. The strong band at 2937 cm^{-1}. established the presence of CH_2 asymmetric stretching. The bands at 884 cm^{-1} established the presence of substituted ring 1,4 distribution. It confirmed that polymerization was successfully done and L-Phe were placed toward the SPR sensor surfaces [2].

References:

1. Akgönüllü, S.; Yavuz, H.; Denizli, A. Preparation of imprinted cryogel cartridge for chiral separation of L-phenylalanine, *Artif Cells Nanomed Biotechnol* **2017**, 45, 800–807.
2. Mahalakshmi, R.; Jesuraja, S.X.; Jerome Das, S. Growth and characterization of L-phenylalanine, *Cryst. Res. Technol* **2006**, 41, 780 – 783.
